# Network pharmacology and experimental evidence of the anti-colorectal cancer potential of Xian Lian Jie Du formula: quercetin, kaempferol and luteolin jointly inhibit the HIF1A/IL6/TNF oncogenic pathway

**DOI:** 10.3389/fonc.2026.1882054

**Published:** 2026-08-31

**Authors:** Ning Du, Li Ji, Lixing Liu, Xinqi Liu, Zhenglong Jiang, Yukun Yin, Li Feng

**Affiliations:** 1 National Cancer Center/National Clinical Research Center for Cancer/Cancer Hospital, Chinese Academy of Medical Sciences and Peking Union Medical College, Beijing, China; 2 Xinglin College of Liaoning University of Traditional Chinese Medicine, Shenyang, China

**Keywords:** cancer toxin framework, colorectal malignancy, molecular docking, network pharmacology, therapeutic interventions, Xian Lian Jie Du formula

## Abstract

Colorectal cancer (CRC) is still one of the main reasons for death from cancer worldwide. Xian Lian Jie Du decoction (XLJD) is based on Professor Zhou Zhongying’s idea of “tumour toxins”, and it has shown some therapeutic effects in CRC; however, its active ingredients and specific molecular targets are not yet fully known. Active constituents of XLJD were screened from the TCMSP database (oral bioavailability ≥30%, drug-likeness ≥0.18). Predicted targets were cross-referenced with CRC-related genes from GeneCards, OMIM, and TTD, and a protein-protein interaction network was built to identify hub targets, which were then annotated using GO and KEGG enrichment. Quercetin, Kaempferol, Luteolin and some other compounds were chosen for molecular docking and 100-ns molecular dynamics simulations. LC-MS was used to identify the XLJD formulation, and then CCK-8 assays and RT-qPCR were carried out to determine the changes in gene expression in HT-29 cells. A total of 197 shared targets were found in XLJD and CRC. Topological evaluation shows that HIF1A, IL6, JUN, MMP9 and TNF are important genes, and most of them belong to the TNF, IL-17 and AGE-RAGE signalling pathways. All fifteen ligand-protein complexes had a docking energy below (more negative than) -5.0 kcal/mol, and TNF was one of them; Molecular dynamics also showed that it had a good binding affinity and formed continuous hydrogen bonds and a stable low-energy state. LC-MS was used to identify the three kinds of compounds: quercetin, hesperidin and luteolin. A large amount of XLJD and every three flavonoids significantly reduced the mRNA expression of the five hub genes in HT-29 cells; however, the low-concentration group had little to no effect. In summary, XLJD and some of its main flavonoids may be targeting a group of CRC-related genes that are controlled by HIF1A, IL6, JUN, MMP9, TNF, etc. The above are the first transcriptional data; protein-based and *in vivo* verification will still be needed.

## 1 Introduction

Colorectal cancer (CRC) is the third most common type of cancer in the world and the second leading reason for death from cancer; it represents a serious problem of malignant diseases around the world (1, 2). Although considerable progress has been made in surgical methods, chemotherapy and targeted therapy, still many people with advanced or relapsed diseases are unable to obtain good outcomes due to tumour recurrence, development of drug resistance, and side effects of long-term therapy (3, 4). Therefore, there is still a need for additional treatment methods that can improve the current protocol and reduce damage to the whole body.

The application of Traditional Chinese Medicine (TCM) in the prevention and treatment of cancer has a history of more than 2,000 years. The foundation of the concept is the Huangdi Neijing, and among the ideas in this book, “zhi wei bing” is considered preventive medicine. At the core of this view is the belief that if the original qi can be properly protected within the body, harmful substances will not be formed, and the accumulation of such substances is often attributed to a deficiency in qi (5). Based on the above philosophy, our research group has used its many years of clinical practice to integrate the concept of “cancer toxin” (ai du) proposed by the famous TCM scholar Professor Zhou Zhongying and constructed a pathomechanistic structure for treating colorectal cancer. We put forward that the basic pathomechanism of CRC relapse is “spleen qi deficiency with stagnation of damp-heat, blood stasis, and renewed reactivation of cancer toxin”, and thus the main therapeutic goals are to strengthen spleen qi, resolve stasis and disperse accumulation, purge heat and drain dampness, and counteract cancer toxin. Based on the above, the formula of Xian Lian Jie Du (XLJD) was formed by adding Astragalus membranaceus (Huangqi), Atractylodes macrocephala (Baizhu), Sparganium stoloniferum (Sanleng), Curcuma zedoaria (Ezhu), Coix lacryma-jobi (Yiyiren), Coptis chinensis (Huanglian), Sophora flavescens (Kushen) and Agrimonia pilosa (Xianhecao) (6).

More and more scientific data have shown that the primary reason for the therapeutic effects of traditional Chinese medicine (TCM) formulations is a change in multiple targets and pathways, and this view aligns with the core idea of network pharmacology in systems biology (7). Quercetin, Kaempferol and Luteolin are all flavonoids that are present in the components of XLJD, and they have been individually shown in experiments to have anti-cancer effects, such as inhibiting cell proliferation, inducing apoptosis and restricting metastasis (8, 9). However, the particular molecular basis of how XLJD can regulate the network of CRC has not been precisely determined yet. To know the reasons for XLJD under the background of rational medical application, we need to reveal its hidden mechanisms and also identify the specific molecular sites that cause this effect. Solving this problem is in line with the current direction of creating new oncological drugs based on mechanisms.

Network pharmacology, molecular docking and cell experiments were used in this paper to study the anti-CRC effect of XLJD. First, a network was constructed to link the medicinal ingredients of XLJD with their potential targets, and then primary active molecules and all hub proteins related to CRC could be identified. Then, to know how the three main flavonoids bind to the five target proteins, molecular docking was used. Later, in order to know if the above interactions had an effect on the body, changes in the mRNA of the target gene in HT-29 cells after treatment with RT-qPCR were determined. Based on the above results, it can be concluded for the first time that specific molecular sites and transcriptional changes may be responsible for the anti-CRC effect of XLJD; however, more functional and *in vivo* studies still need to be conducted before drawing a final conclusion.

## 2 Materials and methods

### 2.1 Network pharmacology

#### 2.1.1 Identification of XLJD active components and targets

The botanical ingredients of the Xian Lian Jie Du prescription (XLJD), that is to say Astragalus membranaceus (Huangqi, 15g), Atractylodes macrocephala (Baizhu, 12g), Sparganium stoloniferum (Sanleng, 10g), Curcuma zedoaria (Ezhu, 10g), Coix lacryma-jobi (Yiyiren, 15g), Coptis chinensis (Huanglian, 10g), Sophora flavescens (Kushen, 10g) and Agrimonia pilosa (Xianhecao, 12g), were searched in the Traditional Chinese Medicine Systems Pharmacology Database and Analysis Platform (TCMSP; https://tcmsp-e.com/). Compounds meeting the thresholds of oral bioavailability (OB) ≥30% and drug-likeness (DL) ≥0.18 were retained, and those lacking corresponding gene names were excluded. Following removal of duplicates, 229 active components remained across the eight constituent herbs. The number of components and corresponding targets identified for each herb was as follows: Huangqi (17 components, 400 targets), Baizhu (4, 22), Sanleng (5, 120), Ezhu (1, 20), Yiyiren (5, 42), Huanglian (11, 256), Kushen (24, 403), and Xianhecao (5, 279).

#### 2.1.2 Construction of herb-component-target network

The 229 identified molecules and their predicted receptors were imported into Cytoscape 3.10.0 (https://cytoscape.org/) to construct a graph of herb compounds and receptors. The following topological evaluation was performed on this structure, and then every node was ranked according to its Degree score. Quercetin, kaempferol, luteolin, formononetin and hederagenin had the highest Degree scores among the vertices and were chosen as the potential key ingredients of XLJD for further investigation.

#### 2.1.3 Colorectal cancer disease target collection

GeneCards, OMIM and the Therapeutic Target Database (TTD) were used to query for “colorectal cancer” in the three databases, and then the genes associated with CRC were obtained. First, the first 50,000 entries of GeneCards were filtered to keep only those with a relevance score of more than 5, and thus 8,848 potential targets were obtained (https://www.genecards.org/). An extra 503 genes were obtained from OMIM (https://www.omim.org/), and 97 were obtained from TTD (https://db.idrblab.net/ttd/). Merge the three collections, remove duplicates, and then 9,184 new unique CRC-linked genes were obtained for further study.

#### 2.1.4 Identification of drug–disease intersecting targets and Venn diagram

Venny 2.1.0 (https://bioinfogp.cnb.csic.es/tools/venny/) was employed to find the common genes of XLJD and CRC, and the results are shown in the following Venn diagram. The 197 usual objects of evaluation were then used to investigate the development and enrichment of the network.

#### 2.1.5 Protein–protein interaction network analysis

The 197 intersecting genes were sent to the STRING platform (https://string-db.org/- only for Homo sapiens, minimal interaction threshold set to 0.400) to generate a PPI map, and this map had 195 vertices and 4,301 links. Then the derived network data were exported to be drawn as graphs in Cytoscape 3.10.0. The topological indices employed in this paper are Degree centrality (DC), Betweenness centrality (BC), Closeness centrality (CC), Eigenvector centrality (EC), Local Average Connectivity (LAC) and Network centrality (NC), all of which were obtained using CytoNCA. Genes that have exceeded the median in all six indices (BC > 57.54, CC > 0.529, DC > 38, EC > 0.051, LAC > 26.23, NC > 28.27) were selected as the first group of hub genes and were numbered from 1 to 72. Then, the 72 molecules were used to construct an improved sub-network, and the same six-parameter screening was carried out again with stricter criteria (BC > 13.93, CC > 0.816, DC > 55, EC > 0.120, LAC > 47.04, NC > 50.58) to reduce the list to 33 essential nodes. According to the centrality of the genes, JUN, MMP9, IL6, HIF1A and TNF were among the first five to be ranked.

#### 2.1.6 GO and KEGG enrichment analyses

Functional profiling of the 197 intersecting candidates was carried out using Gene Ontology (GO) categorization and Kyoto Encyclopedia of Genes and Genomes (KEGG) pathway analysis, and the analysis was performed in RStudio with the clusterProfiler, enrichplot, ggplot2, pathview, ggnewscale and DOSE libraries. Use the org.Hs.eg.db resource to convert the Gene tag into an Entrez ID. The results of the enrichment are significant at P < 0.05, and the q-value threshold has been set to 1. For each GO class (Molecular Function, Cellular Component and Biological Process), the top eight terms were kept, along with the top 20 most important KEGG pathways; both were sorted by P value, and this data was shown in bubble plots, bar charts, network diagrams and circle plots.

### 2.2 Molecular docking

#### 2.2.1 Preparation of ligand structures

The 3D structures of Quercetin (PubChem CID: 5280343), Kaempferol (CID: 5280863) and Luteolin (CID: 5280445) were downloaded from the PubChem database at https://ftp.ncbi.nlm.nih.gov/pubchem/Compound/CURRENT-Full/SDF/ SDF format. All the structures have been optimised to minimise energy, and then AutoDockTools (version 1.5.7) is used to add polar hydrogen atoms, assign Gasteiger partial charges, collapse non-polar hydrogen atoms, and finally convert them into PDBQT format (10).

#### 2.2.2 Preparation of receptor structures

The three-dimensional structures of JUN, MMP9, IL6, HIF1A, and TNF were retrieved from the RCSB Protein Data Bank (PDB, https://www.rcsb.org/), with the following accession codes: HIF1A (PDB: 4H6J), IL6 (PDB: 1ALU), JUN (PDB: 1JNM), MMP9 (PDB: 1L6J), and TNF-α (PDB: 1TNF). Before docking, remove the co-crystallised ligand, solvent molecules and other non-standard residues from all the structures. If there were missing residues or side chains, they could be added with MODELLER or Swiss-PdbViewer. Finally, the receptor was protonated at pH 7.4, energy minimisation was carried out, and then AutoDockTools was employed to convert it to the PDBQT format for docking.

#### 2.2.3 Molecular docking procedure

Molecular docking was carried out with AutoDock Vina 1.1.2 (11). A cubic grid box of 25 x 25 x 25 Å was used for each target, and it was placed either in the centre of the co-crystallised ligand or, in the case of ligand-free structures (4H6J, 1ALU, 1JNM, 1L6J, 1TNF), at the active/binding site indicated by literature and structural annotations (such as UniProt functional site records). Set the exhaustiveness to 8, and then all nine candidate binding modes for the docking simulation will be generated; leave all other parameters as default. Each of the five target proteins was docked individually with all the ligands in this way.

#### 2.2.4 Analysis and visualization of docking results

Among all the structures of a given ligand-receptor pair, the one with the lowest calculated binding free energy was selected as the most stable and thus chosen as the representative. PyMOL 2.x and BIOVIA Discovery Studio Visualizer were used to observe the hydrogen bonds, hydrophobic interactions, van der Waals forces, etc. of the complexes more closely. In line with common practice, a binding energy below (more negative than) -5.0 kcal/mol was taken to indicate favourable ligand-protein binding.

### 2.3 Molecular dynamics

GROMACS 2025.3 was employed to carry out the molecular dynamics simulation of the protein-ligand complex (12). All of them are cubic cells with periodic boundary conditions and are in TIP3P water; the protein is modelled according to AMBER14SB (13). Quantum chemical calculations were carried out in ORCA 6.0 to obtain the structure and partial charge distribution of the ligand (14, 15). The two parts of the calculation are as follows: First, optimise the structure of the gas phase using the B97-3c composite functional, and then single-point energy calculations are carried out with B3LYP, D3 dispersion correction and the def2-TZVP basis set to obtain more reliable electronic distributions. The computation is simple and it is still quite accurate; thus, the RIJCOSX approximation was selected. The molecular electrostatic potential obtained from the above method was used to fit the restrained electrostatic potential charges with Multiwfn (16, 17), and then ligand topologies were generated by Sobtop.

Sodium and chloride counterions were introduced to reproduce a physiological ionic strength of 150 mM. Energy minimisation was performed first, using the conjugate gradient method for 500 steps until a convergence threshold of 100.0 kJ·mol^-^¹·nm^-^¹ was reached, with the maximum step size limited to 0.01 nm. Then, a constrained equilibration period of 100 ps was carried out with a 1 fs time step, and then a 100 ns production phase was run at a 2 fs time step. Set the coupling constant for the V-rescale thermostat to 0.2 ps to set the temperature to 298.15 K, and use coupling constants of 0.5 ps and 2.0 ps for the C-rescale barostat to stabilise the pressure at 1.0 bar. All hydrogen-containing bonds are in the range of LINCS algorithm. The long-range electrostatic interaction was calculated by means of the particle mesh Ewald method at a limit of 1.0 nm, and the same 1.0 nm limit was used for the Verlet scheme in the van der Waals force calculation; dispersion corrections were applied to both the energy and pressure calculations. Centre-of-mass motion was not taken into account in the simulation, and at the production stage, the protein and ligand were given different temperature groups; similarly, angular momentum was also omitted.

Several parameters were extracted from the resulting trajectories: root mean square deviation of the protein Cα backbone, radius of gyration, per-residue root mean square fluctuation, solvent accessible surface area, and the count of protein-ligand hydrogen bonds. Both the 2D and 3D Free Energy Landscapes are based on the reaction coordinate of radius of gyration and root-mean-square deviation, and are then used to study conformational dynamics. The areas shaded in dark blue on this map have relatively low energy, and the structure of these areas has been verified by many simulations.

### 2.4 LC–MS analysis

Disperse the lyophilised XLJD powder in methanol, and then centrifuge to separate the solid residue. Then the obtained supernatant was treated and then analysed by liquid chromatography-mass spectrometry. The initial characterization of some representative components, such as quercetin, hesperidin and luteolin, was carried out by correlating the precursor ion peaks and some fragment ions with information in the existing references.

### 2.5 Cell biology experiments

#### 2.5.1 Reagents and materials

All reagents, cell lines, and consumables are listed in **Table 1**.

**Table 1:** Reagents, cell line, and key materials used in this study.

Reagent/material	Supplier	Catalog no.	Specification
XLJD herbal decoction pieces	Beijing Tongrentang	—	Eight-herb formula, prescribed doses
Quercetin	MedChemExpress (MCE)	HY-18085	Purity ≥ 99.80%
Kaempferol	MedChemExpress (MCE)	HY-14590	Purity ≥ 99.92%
Luteolin	MedChemExpress (MCE)	HY-N0162	Purity ≥ 99.51%
CCK-8 Kit	MedChemExpress (MCE)	HY-K0301	—
McCoy’s 5A medium	Procell	PM150710	—
Foetal Bovine Serum	Excell Bio	FSP500	—
Penicillin/Streptomycin	Procell	PB180120	100×
0.25% Trypsin-EDTA	Procell	PB180225	—
TRIzol^®^ Reagent	Thermo Fisher	15596026	—
HiScript III RT SuperMix	Vazyme	R323-01	—
ChamQ SYBR qPCR Master Mix	Vazyme	Q711-02	—
DMSO (cell-culture grade)	Sigma-Aldrich	D2650	—
PBS (pH 7.4)	Procell	PB180327	—

#### 2.5.2 Cell line and culture conditions

HT-29 human colon adenocarcinoma cells (Catalog No. (CL0118) were obtained from Wuhan Pricella Biotechnology Co., Ltd. (Wuhan, China) and kept in McCoy’s 5A medium with 10% foetal bovine serum and 1% penicillin-streptomycin at 37 °C in a 5% CO_2_ humidified environment. When the cell density in the well reaches 80% - 90%, add 0.25% trypsin-EDTA solution to release the cells, then resuspend and pass. Cells from passages 5 to 15 were used in all the experiments of this study.

#### 2.5.3 Preparation of XLJD lyophilized powder stock solution

Raw crude pieces of the eight medicinal ingredients of XLJD were bought from Beijing Tongrentang in Beijing, China, and then mixed according to the specified ratio. The two stages of extraction are as follows: First, for 60 minutes, eight parts of water are used to boil the blend; Then, for 45 minutes, six parts of water are added and another boiling is carried out. Then, the combined extracts were concentrated under vacuum and freeze-dried to obtain the final XLJD powder (18).

Add a small amount of the lyophilisate to sterile PBS (pH 7.4) for dissolution, and the final concentration should be 20 mg/mL. 2 minutes of vortex mixing was carried out to homogenise the solution, and then ultrasonic treatment (40kHz, 10 minutes, room temperature) was performed. The resulting liquid was then spun at 4000g for 10 minutes at 4 °C, and the clarified fluid was collected. A 0.22 µm PES syringe filter (Millipore) was then used to carry out sterile filtration in a Class II biological safety cabinet according to sterile procedures. To prevent pH-toxicity, the pH of the filtrate was measured with a standard pH meter, and then 0.1M NaOH or 0.1M HCl was added according to need to adjust it to 7.2-7.4. Prepare a 20 mg/ml stock solution, divide it into single doses, and store at -80 °C; a new batch of the working solution must be made immediately before use from the whole culture medium, and any unused portion after a single experiment should be disposed of rather than refrozen.

#### 2.5.4 Preparation of bioactive monomer solutions

Quercetin (MCE, HY-18085, purity ≥99.80%), kaempferol (MCE, HY-14590, purity ≥99.92%), and luteolin (MCE, HY-N0162, purity ≥99.51%) were dissolved separately in cell culture-grade DMSO to prepare 10 mM stock solutions, and then single-use portions were taken from the stock solution and stored at -20 °C in the dark. Before each experiment, a small part was taken from the thawed sample and mixed with the whole medium to prepare the working dilution. At all the treated wells, the final DMSO volume was less than 0.1% (v/v).

#### 2.5.5 CCK-8 cell viability assay

##### 2.5.5.1 XLJD lyophilized powder

In the stage of exponential growth, 1x10^4^ cells per well (100 µL per well) of HT-29 cells were seeded in 96-well plates and left to adhere overnight. To reduce the effect of the edge, 100 µL of sterile PBS was added to the outer well instead of cells. On the following day, the media in the experimental wells were changed to fully supplemented media containing XLJD at concentrations of 0.125, 0.25, 0.5, 1.0, 2.0, 4.0 and 8.0 mg/mL, and each concentration was taken in triplicate; a negative control group (medium + CCK-8 reagent, no cells) and a normal control group (cells cultured in full medium alone) were also added. After 24 h of cultivation at 37 °C with 5% CO_2_, 10 μL of CCK-8 reagent was added to each well, and then the plates were incubated for 1–2 hours at 37 °C in the dark. 450 nm Absorbance (OD_450_) was determined by a microplate reader (Thermo Scientific Multiskan FC). The following are the Cell Viability tests:


$$Cell\;Survival\;\left(\%\right)\;=\;\left(\left(OD\_treatment\;-\;OD\_blank\right)/\left(OD\_control\;-\;OD\_blank\right)\right)\;*\;100$$

Nonlinear regression was used to calculate the IC50 values (log[inhibitor] vs. normalised response) in GraphPad Prism 10.0. XLJD-L and XLJD-H were set as IC_50_/4 and IC_50_/2, respectively.

##### 2.5.5.2 Bioactive monomers

HT-29 cells were treated with Quercetin, Kaempferol, Luteolin and the concentrations of 5, 10, 20, 40, 80 and 100 µM for 24 hours (triplicates); the same operations were performed for all. As in the above, IC50 values were determined. For subsequent qPCR experiments, the working concentration of each monomer was set at a sub-toxic level maintaining ≥85% cell viability, that is below IC_20_, so that transcriptional effects could be assessed with minimal cytotoxic confounding.

#### 2.5.6 Cell treatment and experimental grouping

The cultivation parameters for HT-29 cells were based on the CCK-8 data, and they are shown in **Table 2**. 3x10^5 cells were grown overnight in 6-well plates for the next gene expression experiment to adhere, and then after 24 hours, the treatment was added. There were 3 independent biological replicates (n=3) per single run.

**Table 2:** Experimental grouping for gene expression analysis.

Group	Treatment	Concentration	Vehicle
Control	Complete McCoy’s 5A only	—	—
XLJD-L	XLJD powder, low dose	IC_50_/4 (mg/mL)	Complete medium
XLJD-H	XLJD powder, high dose	IC_50_/2 (mg/mL)	Complete medium
Quercetin	Quercetin monomer ≥ 99.80%	Sub-toxic conc. (μM)	DMSO→medium (≤0.1%)
Kaempferol	Kaempferol monomer ≥ 99.92%	Sub-toxic conc. (μM)	DMSO→medium (≤0.1%)
Luteolin	Luteolin monomer ≥ 99.51%	Sub-toxic conc. (μM)	DMSO→medium (≤0.1%)

#### 2.5.7 RNA extraction and quantitative real-time PCR

##### 2.5.7.1 Total RNA extraction

24 hours after inoculation, wash the cells twice in ice-cold PBS and then lyse them with 1ml of TRIzol^®^ Reagent (Thermo Fisher Scientific, Cat.). No. 15596026 per well. Then, 1.5 mL of RNase-free tubes were taken and kept at room temperature for 5 minutes. Add 200 µl of chloroform, then vigorously vortex the tubes for 15 seconds, incubate for 3 minutes, and finally centrifuge at 12,000 times g for 15 minutes at 4 °C. The top part was transferred to a new tube, and then to carry out RNA purification by adding 500 µl of isopropanol and leaving it at room temperature for 10 minutes. Centrifuge (12,000 × g for 10 minutes at 4 °C) to obtain the RNA pellet; then, wash the RNA pellet twice with 75% ethanol, dry it, and finally resuspend in RNase-free water. Nanodrop spectrophotometry was used to determine the quantity and quality of the extracted RNA, and only the samples with an A260/A280 ratio between 1.8 and 2.1 met the requirements for the next step.

##### 2.5.7.2 cDNA synthesis

500 ng of total RNA per sample was used to synthesize cDNA with the help of HiScript III RT SuperMix reagent (Vazyme, Cat.). No. R323-01) according to the supplier’s instructions (37 °C, 15 min; 85 °C, 5 s). Dilute the obtained cDNA with RNase-free distilled water 1:5 and then store it at -20 °C.

##### 2.5.7.3 Quantitative real-time PCR

ChamQ Universal SYBR qPCR Master Mix (Vazyme, Cat.) was used for the RT-qPCR assay. No. (Q711-02) According to the data from the PR-96 real-time PCR instrument (Hangzhou Miao Instruments Co., Ltd., Hangzhou, China). Add 20 µL of the mixture, then add 10 µL of 2× SYBR Master Mix, 0.4 µL of both the forward and reverse primers (at a concentration of 10 μM), 2 µL of the cDNA sample, and finally 7.2 µL of RNase-free water. The program for the thermal cycle is as follows: First, keep at 95 °C for 30 s; then, for 40 cycles, raise the temperature to 95 °C for 10 s and at the same time reduce it to 60 °C for 30 s; finally, perform melt curve analysis (65-95 °C, 0.5 °C step, 5 s per step) to confirm the amplification specificity. All the experiments were done three times technically.

The relative mRNA levels of the five genes (JUN, MMP9, IL6, HIF1A and TNF) were expressed in terms of the internal standard gene β-actin (ACTB)by means of the 2−ΔΔCt method. All the primer sets are human-specific and designed to cross the exon-exon boundary to avoid the amplification of genomic DNA; they are listed in **Table 3**.

**Table 3:** Human-specific RT-qPCR primer sequences used in this study.

Gene	Direction	Primer sequence (5′→3′)	Amplicon
ACTB (Reference)	Forward	GAAGGTGAAGGTCGGAGTC	226 bp
	Reverse	GAAGATGGTGATGGGATTTC	
JUN	Forward	CGCATGAGGAACCGCATCG	118 bp
	Reverse	TTGAGCGTGGTTTTTCTGCC	
MMP9	Forward	TGTACCGCTATGGTTACACTCG	182 bp
	Reverse	GGCAGGGACAGTTGCTTCT	
IL6	Forward	AGACAGCCACTCACCTCTTCAG	197 bp
	Reverse	TTCTGCCAGTGCCTCTTTGCTG	
HIF1A	Forward	TATGAGCCAGAAGAACTTTTAGGC	151 bp
	Reverse	CACCTCTTTTGGCAAGCATCC	
TNF	Forward	CAGCCTCTTCTCCTTCCTGAT	211 bp
	Reverse	GCCAGAGGGCTGATTAGAGAG	

All primers are designed for Homo sapiens sequences, validated for SYBR Green-based RT-qPCR at 60 °C annealing temperature. Amplicon specificity was confirmed by melt-curve analysis. ACTB was used as the internal reference gene.

#### 2.5.8 Statistical analysis

The mean ± standard deviation are shown in the three autonomous biological replicates (n=3) per trial of the numerical results. GraphPad Prism 10.0 (GraphPad Software, San Diego, CA, USA) was used for the statistics; a one-way ANOVA was conducted on the differences among the various groups of students, and then a Tukey’s *post-hoc* test was performed; P < 0.05 was taken as the statistical significance level. The signs of the significance markers are as follows: *P < 0.05, **P < 0.01, ***P < 0.001, ****P < 0.0001, ns = not significant.

## 3 Results

### 3.1 Network pharmacology analysis

#### 3.1.1 XLJD active components and herb–component–target network

**Figure 1:**
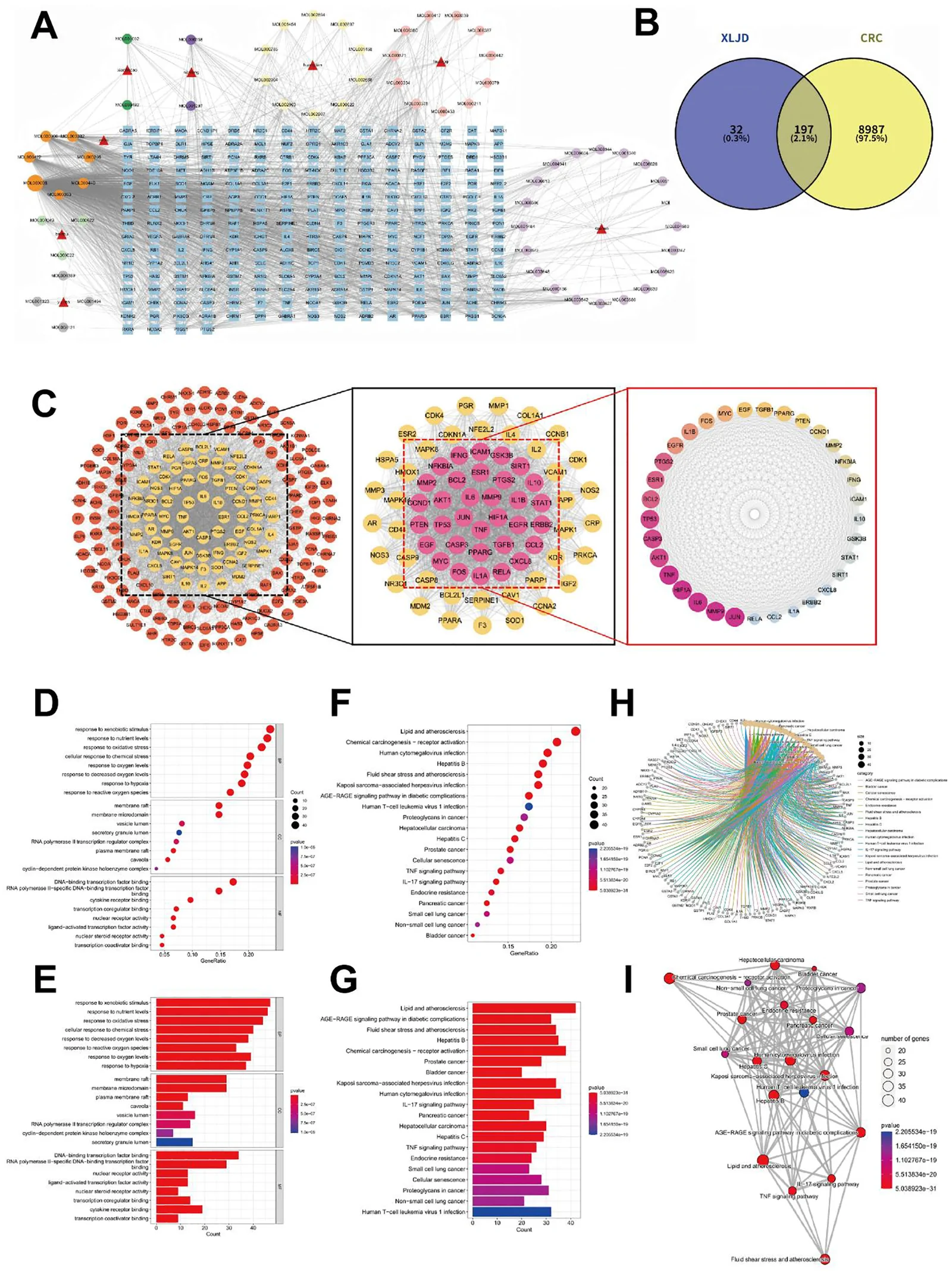
Network pharmacology analysis of XLJD in colorectal cancer. **(A)** Herb–component–target network constructed in Cytoscape 3.10.0. Orange circles represent herbs, triangles represent active components, and blue rectangles represent target proteins. Node size is proportional to Degree value. **(B)** Venn diagram illustrating the overlap between XLJD component targets (n = 229) and colorectal cancer disease targets (n = 9,184), yielding 197 intersecting targets. **(C)** Protein–protein interaction (PPI) network of the 197 intersecting targets generated using the STRING database and visualized in Cytoscape. Nodes are coloured by centrality, with the first-round filtered subnetwork shown in the magnified centre panel and the secondary filtered subnetwork containing 33 core targets shown on the right; node size reflects Degree value. **(D)** Bubble plot depicting Gene Ontology (GO) enrichment results, with the eight most enriched terms shown for each of the Biological Process (BP), Cellular Component (CC), and Molecular Function (MF) categories; bubble size reflects gene count, and colour denotes adjusted P value. **(E)** Bar chart presenting the same GO enrichment results (top eight terms per category for BP, CC, and MF), where bar length corresponds to gene count and colour indicates adjusted P value. **(F)** Bubble plot summarising KEGG pathway enrichment analysis, displaying the 20 most significantly enriched pathways; bubble size reflects gene count, and colour denotes adjusted P value. **(G)** Bar chart of the same KEGG enrichment results, with the top 20 significantly enriched pathways shown; bar length corresponds to gene count, and colour indicates adjusted P value. **(H)** Chord diagram depicting associations between target genes and the significantly enriched KEGG pathways. **(I)** Network diagram showing relationships among the significantly enriched KEGG pathways, in which node size represents gene count and colour denotes adjusted P value.

A total of 229 good compounds and their corresponding molecular targets have been retrieved from TCMSP for the eight medicinal herbs in XLJD. After importing to Cytoscape 3.10.0, a framework of herb-ingredient-target was constructed, and then the topological analysis of each node was carried out according to the Degree metric. Based on the degree score, the five molecules that appeared in the list of active ingredients for XLJD were quercetin, kaempferol, luteolin, formononetin and hederagenin (**Figure 1A**).

#### 3.1.2 Drug–disease intersecting targets

After the combination and purification of GeneCards, OMIM and TTD, there were 9,184 disease markers for colorectal cancer. The intersection with the 229 XLJD constituent targets is shown in a Venn diagram; the number of common genes is 197 (2.1%), and there are 32 targets exclusive to XLJD and 8,987 condition-specific genes(**Figure 1B**). The 197 overlapping genes were selected as the therapy targets, and then these were used in all the following experiments.

#### 3.1.3 PPI network analysis and hub target identification

A total of 197 intersecting genes were used in STRING to construct a PPI network, and it has 195 nodes and 4,301 edges. Then, the six centrality indicators (DC, BC, CC, EC, LAC, NC) were employed in the two rounds of topological screening in Cytoscape to obtain the top 33 important genes. Among this group, JUN, MMP9, IL6, HIF1A and TNF had the highest importance and were considered to be the five main central targets (**Figure 1C**).

#### 3.1.4 GO and KEGG enrichment analysis

Functional division of the 197 intersection points showed an increase in the three groups. Biological Processes are classified based on how toxicants and oxidative stress affect the inside of cells, changes in cell division, hypoxia adaptation, etc. The labels of the main components of primary cells are lipid rafts, extracellular matrix and exocytic organelles, and the key descriptions of molecular functions are transcription factor interactions, nuclear hormone signalling and cytokine receptor binding (**Figures 1D, E**). A total of 20 KEGG pathways were at the statistical significance level (P < 0.05), and among them, lipid metabolism in atherosclerosis, receptor-dependent chemical oncogenesis, AGE-RAGE cascades, fluid shear stress-induced atherosclerosis, the TNF and IL-17 signalling pathway, etc., were relatively more significant. Many oncogenic pathways were also overrepresented in the same way, such as those in hepatocellular carcinoma, prostate malignancy, bladder cancer and non-small cell lung cancer (**Figures 1F–I**).

### 3.2 Molecular docking

#### 3.2.1 Binding affinity of core active constituents with hub targets

**Figure 2:**
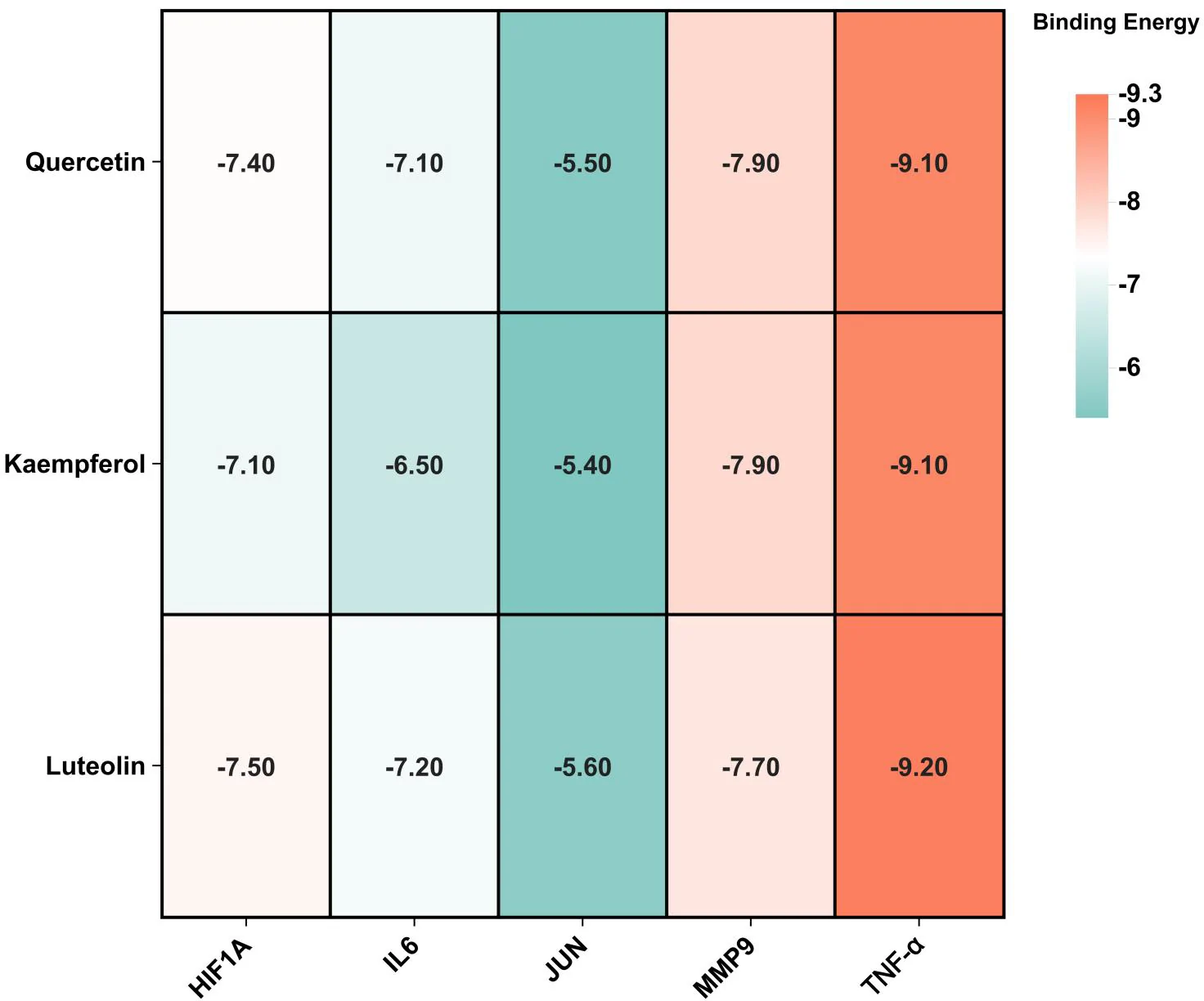
Heatmap of molecular docking binding energies. Heatmap of binding free energies (ΔG, kcal/mol) calculated for all combinations between the three core active constituents (quercetin, kaempferol, luteolin) and five hub target proteins (HIF1A, IL6, JUN, MMP9, TNF-α), with colour ranging from teal (indicating weaker binding) to orange-red (indicating stronger binding). All values are below −5.0 kcal/mol, indicating favourable binding for all 15 pairs.

AutoDock Vina software was used for molecular docking to determine the degree of binding between quercetin, kaempferol, luteolin and HIF1A, IL6, JUN, MMP9 and TNF-α. All fifteen ligand-target pairs gave docking scores below (more negative than) -5.0 kcal/mol, indicating favourable binding in every case (**Figure 2**). TNF-α was the most strongly bound of the five targets, with binding energies of -9.10, -9.10 and -9.20 kcal/mol for quercetin, kaempferol and luteolin, respectively. MMP9 ranked second, ranging from -7.70 to -7.90 kcal/mol (quercetin -7.90, kaempferol -7.90, luteolin -7.70), whereas JUN was the least favourable target, ranging from -5.40 to -5.60 kcal/mol (quercetin -5.50, kaempferol -5.40, luteolin -5.60). Averaged across the five targets, luteolin gave the most negative mean docking energy (-7.44 kcal/mol), followed by quercetin (-7.40 kcal/mol) and kaempferol (-7.20 kcal/mol), indicating comparable overall predicted binding strength among the three flavonoids.

#### 3.2.2 Characterization of binding interaction

**Figure 3:**
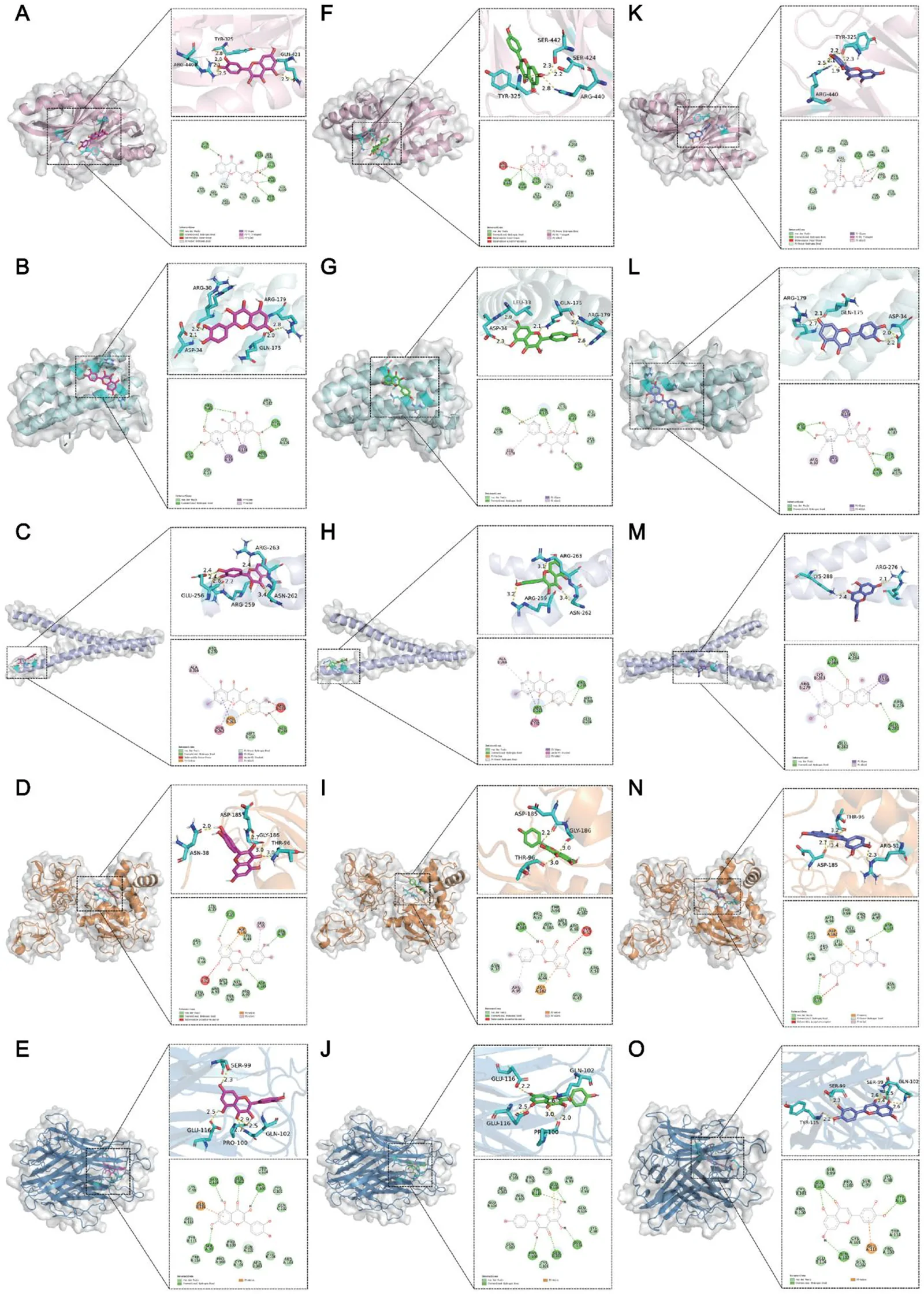
Molecular docking of core active constituents with hub target proteins. Three-dimensional binding poses (top left of each panel) and two-dimensional interaction diagrams (bottom right of each panel) are shown for each ligand–receptor pair. Hydrogen bond distances are indicated in Å. **(A–E)** Quercetin docked to HIF1A, IL6, JUN, MMP9, and TNF-α, respectively. **(F–J)** Kaempferol docked to HIF1A, IL6, JUN, MMP9, and TNF-α, respectively. **(K–O)** Luteolin docked to HIF1A, IL6, JUN, MMP9, and TNF-α, respectively. Key interacting residues are labelled in the 3D panels; interaction types in the 2D diagrams include hydrogen bonds, hydrophobic contacts, and van der Waals interactions.

To understand the nature of the ligand-receptor interaction in all the above complexes, ideal docking models were employed (**Figure 3**). HIF1A has the same binding pattern for all three compounds, and the centre of binding is TYR-325 and ARG-440; quercetin has an extra interaction with GLN-421, kaempferol binds to SER-442 and SER-424, and luteolin is primarily bound by the TYR-325/ARG-440 pair (**Figures 3A, F, K**). As for IL6, ARG-179, GLN-175 and ASP-34 formed a repeating hydrogen-bond ensemble for all three molecules, and quercetin also formed interactions with ARG-30 and kaempferol via LEU-33 (**Figures 3B, G, L**). JUN, quercetin and kaempferol have similar binding patterns with ARG-263, ARG-259 and ASN-262 (and quercetin also binds GLU-256), but luteolin is directed at a different site, LYS-288 and ARG-276; thus, these three flavonoids have different binding orientations in the JUN cavity (**Figures 3C, H, M**). ASP-185 and THR-96 have been consistently involved in MMP9, and quercetin, kaempferol and luteolin have also bound ASN-38, GLY-186 and ARG-51 respectively (**Figures 3D, I, N**). GLN-102 is bound by all three compounds with TNF-α and is one of the main sites for recurrent hydrogen bonding; at the same time, quercetin forms hydrogen bonds with SER-99, GLU-116 and PRO-100; kaempferol binds to GLU-116 and PRO-100; and luteolin has hydrogen bonds with SER-99 and TYR-115 (**Figures 3E, J, O**). Hydrogen bonding combined with hydrophobic interactions were the main types of interactions in all the complexes that had been studied, and this was consistent with the previous results on binding affinity. The list of popular hot-spot residues and their specificity to the five targets is given in the **Supplementary Table**.

### 3.3 Molecular dynamics

**Figure 4:**
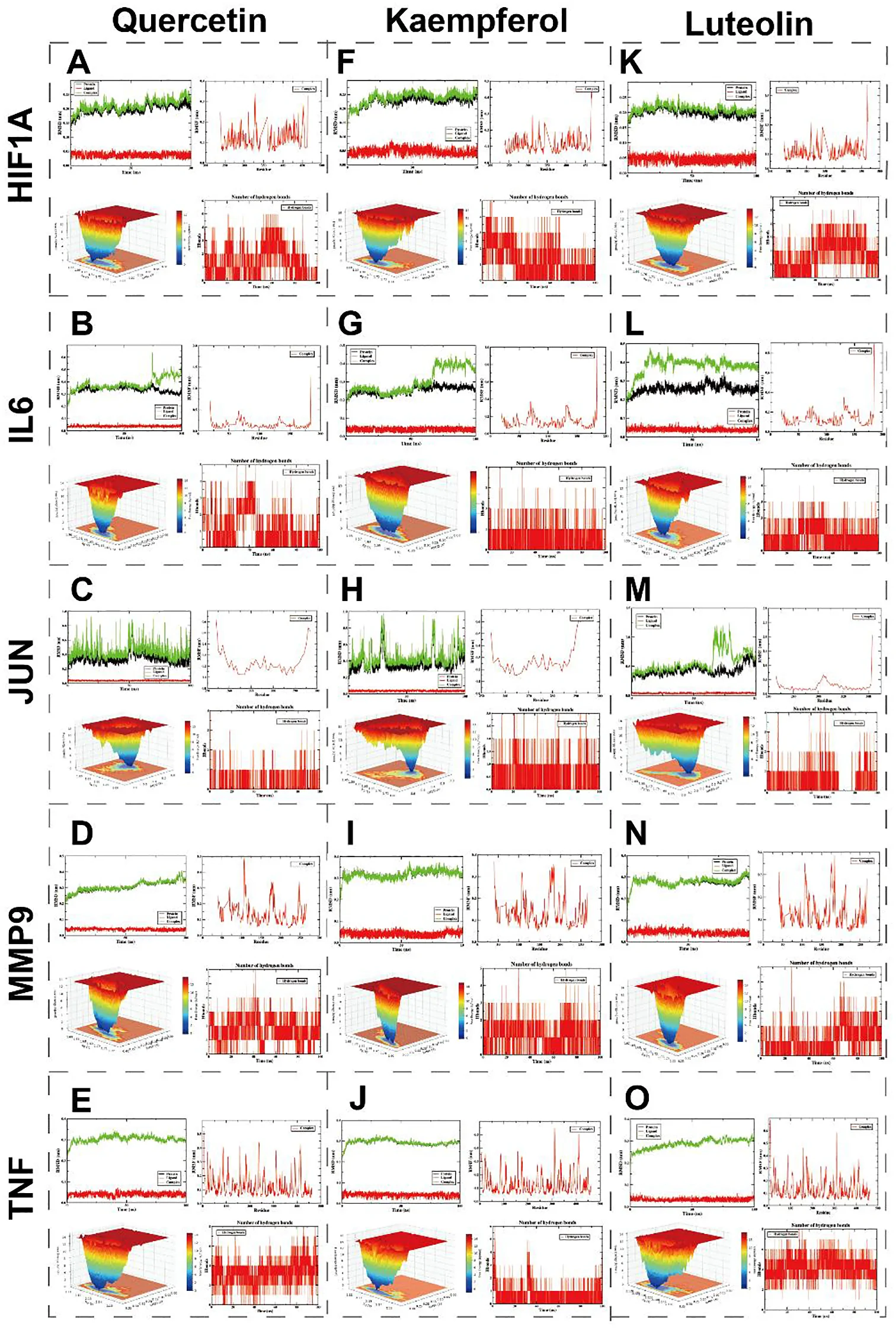
Molecular dynamics simulation analysis of quercetin, kaempferol, and luteolin complexes with five hub targets. **(A–O)** RMSD, RMSF, three-dimensional free-energy landscape, and hydrogen-bond analyses of quercetin, kaempferol, and luteolin complexes with HIF1A, IL6, JUN, MMP9, and TNF during the 100-ns molecular dynamics simulations.

100 ns of molecular dynamics simulation was carried out to study the structure stability of the complex formed by quercetin, kaempferol, luteolin, HIF1A, IL6, JUN, MMP9 and TNF. The RMSD trajectories of all pairs have been stable for some time after a brief period of equilibration and do not change significantly since (**Figure 4**). The Range of Motion was small, as shown in RMSF; most of the large displacements occurred in the relatively free regions of the protein. At different times along the trajectory, there were hydrogen bonds with the ligands and their target binding pockets, and although they were frequently formed, they were transient electrostatic attractions rather than stable bonds. There are three-dimensional free energy maps for all complexes, and they all have a single deep minimum that corresponds to the lowest energy. As shown in **Supplementary Figure 1**, the radius of gyration and solvent-accessible surface area showed only small fluctuations during the calculation; thus, it can be concluded that both the spatial compactness and the aqueous accessibility were well maintained. There were some low-energy regions in the two-dimensional free energy maps locally as well, and therefore the structural stability of the studied system was further verified. Based on the above results, it can be seen that quercetin, kaempferol and luteolin were stably bound to the five key proteins for the whole 100 ns simulation time, although there were some differences in mobility and hydrogen bonding patterns among these complexes.

### 3.4 LC–MS characterization of XLJD

**Figure 5:**
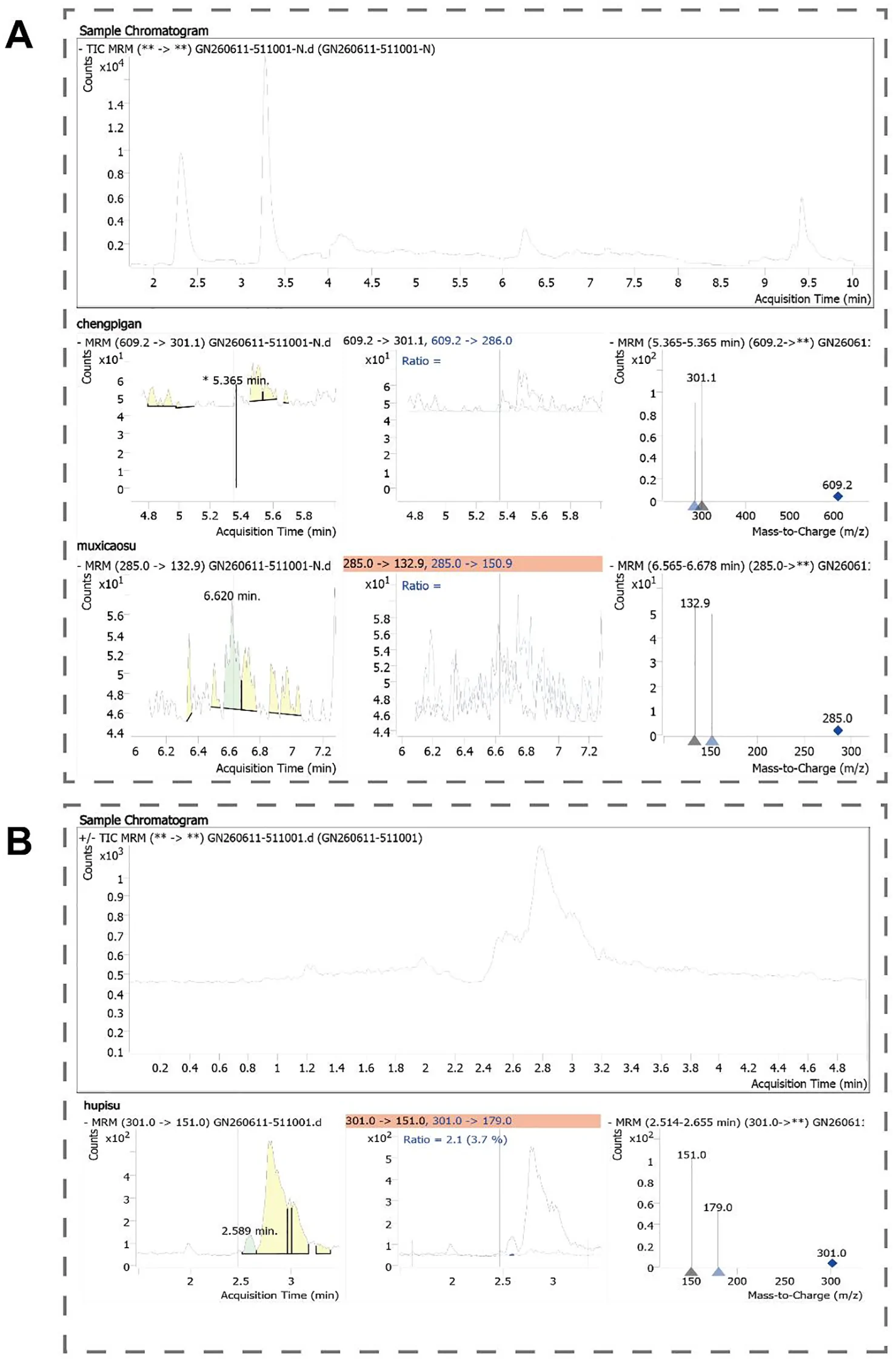
LC–MS/MS analysis of representative active components in XLJD. **(A)** LC–MS/MS chromatograms and characteristic ion signals of hesperidin and luteolin; **(B)** LC–MS/MS chromatogram and characteristic ion signals of quercetin.

LC-MS analysis of the lyophilised XLJD formulation showed some peaks for quercetin, hesperidin and luteolin. The above results have shown that the three constituent parts are indeed present in a qualitative manner and may constitute a part of the structurally-based effect of XLJD (**Figure 5**).

### 3.5 Cell biology results

#### 3.5.1 CCK-8 cell viability and working concentration determination

CCK-8 assay was employed to determine the cytotoxicity and the three types of biological activities of XLJD lyophilisate extract in HT-29 cells after 24 hours of incubation. XLJD had an IC_50_ of 3.82 mg/mL, and therefore XLJD-L and XLJD-H were classified as IC_50_/4 (approximately 0.96 mg/mL) and IC_50_/2 (approximately 1.91 mg/mL), respectively. IC_50_ values for quercetin, kaempferol and luteolin were 98.6 μM, 204.3 μM and 82.7 μM, respectively. The kaempferol IC_50_ lies beyond the highest concentration tested (100 μM, 72.3% viability) and was therefore obtained by extrapolation from the fitted dose-response curve; it should be read as an approximate estimate rather than a directly measured value. Cell morphology images of all treatment groups at their respective IC_50_ concentrations are presented in **Supplementary Figure 2**. Working concentrations maintaining ≥85% cell viability, that is below IC_20_, were selected for the subsequent qPCR experiments: quercetin at 20 μM (92.0% viability), kaempferol at 40 μM (90.9%) and luteolin at 20 μM (89.4%) (**Table 4**). The two XLJD doses were instead defined relative to the XLJD IC_50_ itself, giving XLJD-L at IC_50_/4 (≈0.96 mg/mL, approximately 86% viability) and XLJD-H at IC_50_/2 (≈1.91 mg/mL, approximately 76% viability).

**Table 4:** CCK-8 cell proliferation data of XLJD dried powder and the three bioactive components in HT-29 cells after 24 h.

Compound	Concentration	OD_450_ (mean ± SD)	Cell viability (%)	IC_50_
XLJD lyophilized powder	0.125 mg/mL	0.984 ± 0.031	97.8 ± 3.1	
	0.250 mg/mL	0.961 ± 0.027	95.4 ± 2.7	
	0.500 mg/mL	0.921 ± 0.034	91.3 ± 3.4	
	1.000 mg/mL	0.862 ± 0.028	85.5 ± 2.8	
	2.000 mg/mL	0.755 ± 0.041	74.8 ± 4.1	
	4.000 mg/mL	0.559 ± 0.036	55.4 ± 3.6	IC_50_ = 3.82 mg/mL
	8.000 mg/mL	0.307 ± 0.029	30.4 ± 2.9	
Quercetin	5 μM	1.003 ± 0.022	99.6 ± 2.2	
	10 μM	0.976 ± 0.019	96.9 ± 1.9	
	20 μM	0.927 ± 0.030	92.0 ± 3.0	
	40 μM	0.842 ± 0.035	83.6 ± 3.5	
	80 μM	0.643 ± 0.028	63.8 ± 2.8	IC_50_ = 98.6 μM
	100 μM	0.491 ± 0.033	48.7 ± 3.3	
Kaempferol	5 μM	1.008 ± 0.020	100.1 ± 2.0	
	10 μM	0.994 ± 0.025	98.7 ± 2.5	
	20 μM	0.967 ± 0.033	96.0 ± 3.3	
	40 μM	0.916 ± 0.029	90.9 ± 2.9	
	80 μM	0.815 ± 0.038	80.9 ± 3.8	IC_50_ = 204.3 μM
	100 μM	0.729 ± 0.031	72.3 ± 3.1	
Luteolin	5 μM	0.999 ± 0.021	99.2 ± 2.1	
	10 μM	0.963 ± 0.027	95.6 ± 2.7	
	20 μM	0.901 ± 0.032	89.4 ± 3.2	
	40 μM	0.793 ± 0.030	78.7 ± 3.0	
	80 μM	0.564 ± 0.037	55.9 ± 3.7	IC_50_ = 82.7 μM
	100 μM	0.391 ± 0.029	38.8 ± 2.9	

Working concentrations selected for the qPCR experiments. Monomers were used at concentrations maintaining ≥85% cell viability (below IC_20_): quercetin = 20 μM (92.0%); kaempferol = 40 μM (90.9%); luteolin = 20 μM (89.4%). XLJD doses were defined relative to its own IC_50_: XLJD-L = IC_50_/4 ≈ 0.96 mg/mL (approximately 86% viability); XLJD-H = IC_50_/2 ≈ 1.91 mg/mL (approximately 76% viability). The kaempferol IC_50_ (204.3 μM) exceeds the highest concentration tested (100 μM) and is an extrapolated value.

Mean ± SD (n = 3) are used to present the values. Nonlinear regression in GraphPad Prism 10.0 was used to determine the IC_50_ values.

#### 3.5.2 Effects of XLJD and bioactive monomers on hub gene expression

**Figure 6:**
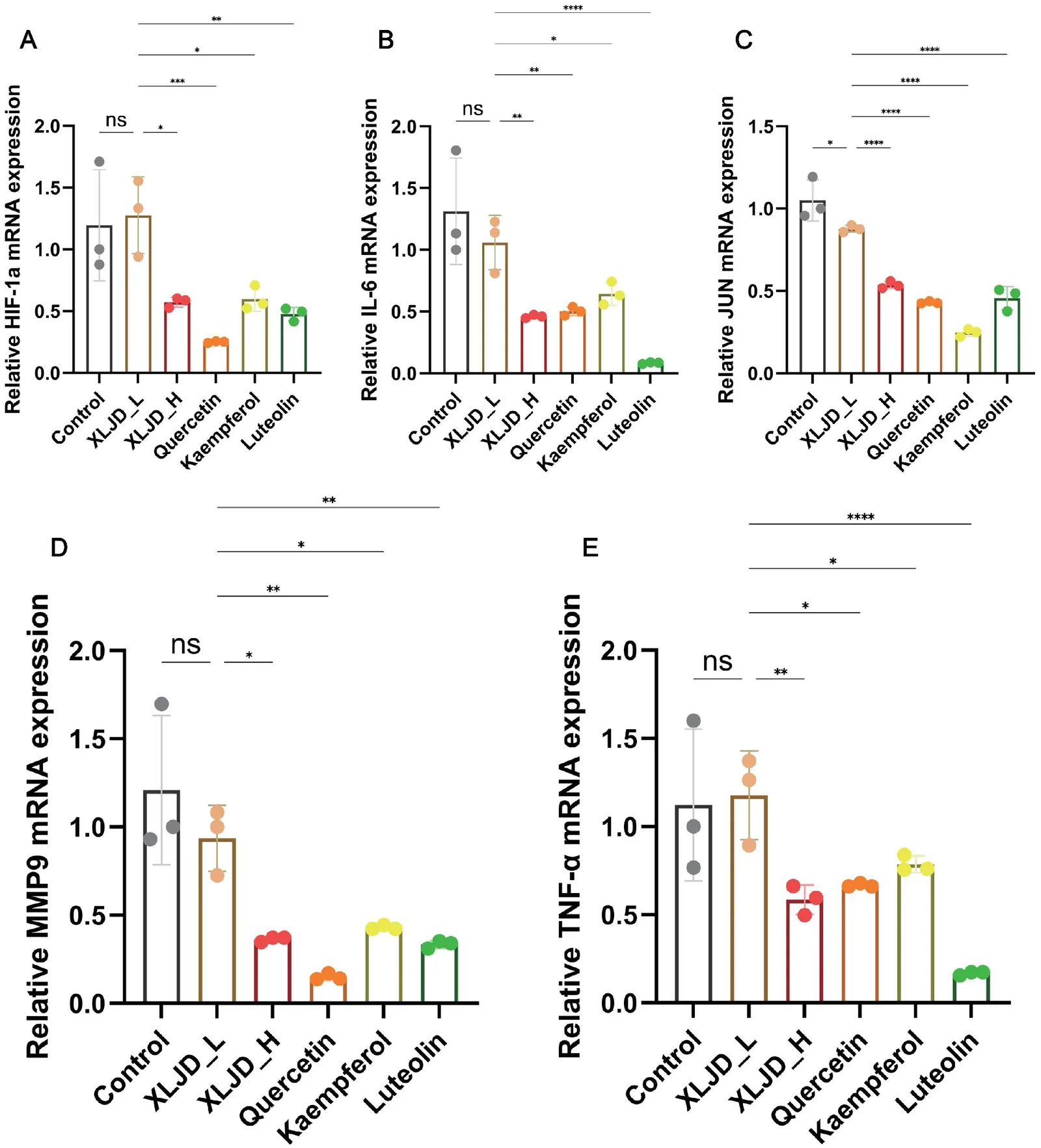
Effects of XLJD and bioactive monomers on hub gene mRNA expression in HT-29 cells. RT-qPCR analysis of **(A)** HIF1A, **(B)** IL6, **(C)** JUN, **(D)** MMP9, and **(E)** TNF-α mRNA levels in HT-29 cells treated for 24 h with XLJD-L (IC_50_/4), XLJD-H (IC_50_/2), Quercetin (20 μM), Kaempferol (40 μM), or Luteolin (20 μM). Expression levels were normalized to ACTB using the 2-ΔΔCt method and are presented as mean ± SD (n = 3). Statistical comparisons were performed by one-way ANOVA with Tukey’s *post-hoc* test. *P < 0.05, **P < 0.01, ***P < 0.001, ****P < 0.0001; ns, not significant.

To know whether XLJD and its three parts can change the mRNA content of the five target genes in HT-29 cells, quantitative real-time PCR will be carried out. Compared with the control group, there was a typical reduction in the amount of HIF1A, IL6, JUN, MMP9 and TNF-α after the intervention. Generally speaking, the modifications were more pronounced in the XLJD-H and single flavonoid groups than in the XLJD-L group (**Figure 6**). To check if the primers have good specificity, melt-curve analysis was carried out, and the typical profiles are presented in **Supplementary Figure 3**.

HIF1A (**Figure 6A**) did not change significantly after XLJD-L, and it was reduced to a considerable extent by XLJD-H (P<0.05). Quercetin, kaempferol and luteolin also reduced the expression of HIF1A (P<0.001, P<0.05, P<0.01 respectively).

IL6 expression was the same as that in **Figure 6B**; XLJD-L did not change significantly, but XLJD-H reduced the amount of IL6 mRNA by more than 50% (P<0.01). Every three flavonoids reduced the output of IL6; among them, luteolin was the most effective (P < 0.0001).

The level of JUN decreased after both XLJD interventions (**Figure 6C**), and among them, XLJD-L had a small effect (P<0.05), and quercetin, kaempferol, and luteolin were all significantly reduced (P<0.0001).

As shown in **Figure 6D**, MMP9 did not change significantly with XLJD-L, but both XLJD-H and each individual monomer significantly reduced the level of MMP9 (P < 0.05, P < 0.01, P < 0.05 and P < 0.01, respectively).

The amount of TNF-α mRNA was the same in the presence of XLJD-L and reduced significantly after XLJD-H treatment (**Figure 6E**). Quercetin, kaempferol and luteolin all decreased in amount, but luteolin was reduced to a greater extent (P<0.0001). Taken together, the above results show that XLJD and the three flavonoids can regulate the transcription of the five hub genes in HT-29 cells. The decrease in the amount of mRNA was not predicted by the change in molecular docking affinity.

## 4 Discussion

Network pharmacology, molecular docking, molecular dynamics simulation and RT-qPCR have been used to study the molecular mechanism of XLJD in colorectal cancer in more detail. HIF1A, IL6, JUN, MMP9, and TNF-α were selected as the main hub molecules, and then they were tested in HT-29 cells. The results of RT-qPCR were in line with the calculations. Nevertheless, this information should be regarded as the mechanism by which anti-tumour drugs work, not as an effect of the drugs on tumours.

XLJD and CRC have combined network pharmacology to get 197 targets so far. Topological evaluation was then used to limit the list to the 33 primary objects. The top five nodes were HIF1A, IL6, JUN, MMP9 and TNF, and they were considered to be in the signal network of CRC. GO and KEGG enrichment analysis show that the genes above are mainly involved in the response of cells to hypoxia and oxidative stress, inflammatory cytokine signalling, transcriptional regulation, extracellular matrix remodelling, etc. The main KEGG pathways are lipids and atherosclerosis (19), AGE-RAGE signalling (20), TNF signalling (21), and IL-17 signalling (22), which have all been individually associated with CRC, and their joint enrichment here suggests that XLJD may simultaneously affect the inflammatory, hypoxic and invasive characteristics of the tumour microenvironment.

Based on previous studies, there are some agreements between the present observations and the pathways of these flavonoids. Kaempferol can trigger apoptosis in SW480 colorectal cancer cells by activating DR4 and DR5 through TRAIL (23), and when there is no CRC, it has also been found that kaempferol interferes with HIF-mediated vascular signals and the ERK-p38-JNK/AP-1 pathway (24, 25). Luteolin has also been shown to inhibit the growth, spread and angiogenesis of cancer cells (26). The above data provide the basis for the existing results, but the published mechanisms are not directly related to the five potential targets, and more verification of quercetin is needed.

The Functions of the five main objects need to be studied further. HIF1A is the symbol for hypoxia-inducible factor 1-alpha, and it is one of the main transcriptional regulators of the oxygen-deprived state in cells (27). HIF1A can promote the formation of tumour blood vessels, metabolic changes in cancer cells, escape from apoptosis, etc., by upregulating genes such as VEGF, LDHA and BCL2 (28), in CRC. Increased expression is associated with the progression of the disease, lymphatic spread and worse clinical outcomes (29). Based on the observations, it is found that XLJD-H plus the three flavonoids can reduce the amount of HIF1A mRNA in HT-29 cells; therefore, it can be concluded that XLJD inhibits the angiogenic and metabolic changes that promote cancer cell growth under hypoxia.

IL6 is involved in the development of CRC and acts through the JAK/STAT3 pathway (30). Overexpression of IL-6 can promote the phosphorylation of STAT3 and thus foster the growth of cancer cells; at the same time, it induces epithelial-to-mesenchymal transition (EMT) and decreases sensitivity to conventional chemotherapy drugs (31). In line with the traditional logic of XLJD, high doses of XLJD or certain flavonoids reduced IL-6 levels. Therefore, it may be that the formula can reduce the signal transduction of pro-inflammatory mediators in the tumour microenvironment to some extent and thus have a heat-clearing and dampness-expelling effect.

JUN belongs to the AP-1 family of transcription factors and is a signal transmitter for cell growth, division and transformation under injury; at the same time, it also promotes spread in cancer (32, 33). There is frequent dysregulation of AP-1 in CRC, which is associated with resistance to EGFR-targeted therapy (34). A small and gradual decrease in the JUN mRNA of XLJD-H and the three monomers may be due to a general reduction in the expression of stress-response genes. JUN showed the weakest binding among the five targets in molecular docking (-5.40 to -5.60 kcal/mol), although all three values remained below (more negative than) the -5.0 kcal/mol threshold for favourable binding. Given the marked transcriptional suppression observed experimentally, secondary pathway-level effects are likely to contribute alongside direct binding.

MMP9 is needed to degrade the extracellular matrix and break down basement membranes; at this time, it limits the spread of cells and blood vessels to different areas and the spread of blood (35). An increase in MMP9 in CRC tissue is an indication of poor prognosis (36). Among the compounds tested, quercetin produced the largest reduction in MMP9 mRNA, lowering expression to approximately 15% of the control level, that is a reduction of about 85% (**Figure 6D**). It should be noted that quercetin and kaempferol were assigned an identical docking score for MMP9 (-7.90 kcal/mol), so the docking results cannot discriminate between the two compounds; the identification of quercetin as the most effective suppressor therefore rests on the RT-qPCR data alone. A plausible explanation lies downstream of direct binding, since quercetin has previously been shown to suppress MMP9 expression by interfering with NF-κB and AP-1 dependent transcription (37). The simultaneous downregulation of JUN and MMP9 mentioned here may be due to an integrated pathway, as AP-1 is a main transcriptional factor for MMP9 (38).

TNF-α has a stronger binding affinity than the other two flavonoids (ΔG: −9.10 to −9.20 kcal/mol), and after luteolin treatment, it was found that the transcription of the receptor was suppressed the most; luteolin also had the highest calculated binding energy (-9.20 kcal/mol). TNF-α has two different effects in cancer, and its levels are low in the tumour microenvironment; at this time, sustained TNF-α signals can activate NF-κB promote cell survival (39), and angiogenesis, but at therapeutic doses, it can induce apoptosis (40). The reduction in the baseline level of TNF-α in HT-29 cells by XLJD and its components indicates that the pro-tumorigenic branch of this signalling network has been impaired; therefore, it is consistent with the KEGG enrichment results of the TNF pathway and the “anti-cancer toxin” therapeutic concept of the herbal formula.

Molecular docking and RT-qPCR are at different levels in biology, but a certain degree of qualitative coherence was observed in the predicted component-target associations and the gene expression patterns of HT-29 cells. TNF-α and MMP9 showed good predicted binding to the evaluated substances, and they also reduced in mRNA after treatment; On the other hand, the JUN reaction was less consistent, although it had a weak predicted interaction. The above results are in line with the proposed component-target links, and it can be concluded that direct ligand-target interaction is a possible path. However, it has not been shown that the degree of mRNA reduction is directly associated with the strength of binding at the docking site, so other regulatory pathways may also be involved.

The quantitative distribution observed by LC-MS/MS warrants explicit consideration. The free aglycone content of quercetin, kaempferol and luteolin in the XLJD preparation was low relative to phenolic acids such as protocatechuic acid and protocatechualdehyde. This observation does not in itself argue against a pharmacological role for the three flavonoids. Abundance in the administered preparation is only one determinant of pharmacological contribution, which depends additionally on intrinsic potency, target relevance and achievable exposure at the site of action. Two considerations are particularly relevant here. First, flavonoids in aqueous decoctions occur predominantly as glycosides, so the measured free-aglycone content underestimates the aglycone pool released *in vivo* through intestinal and microbial β-glucosidase activity following oral administration. Second, protocatechuic acid is a recognised microbial catabolite of flavonoids and anthocyanins, and its abundance in the decoction may partly reflect degradation chemistry rather than an independent active principle. At the same time, both protocatechuic acid and protocatechualdehyde possess documented anti-inflammatory and anti-tumour activity, including the induction of apoptosis in colon cancer cells, and their contribution to the overall activity of XLJD cannot be excluded on the present evidence. The three flavonoids examined here should therefore be regarded as representative candidate constituents selected on network-pharmacological grounds, rather than as the exclusive active principles of the formula. Several limitations of the present study merit acknowledgement. Network pharmacology is a hypothesis-generating approach whose results depend on the quality and coverage of the underlying databases. GeneCards rankings may be influenced by publication volume and research attention, while STRING includes predicted, text-mined, and indirect associations in addition to experimentally validated interactions. TCMSP may be affected by incomplete component information, database updates, and inaccuracies in predicted compound–target relationships, and its fixed OB and DL thresholds may exclude potentially active compounds. Similarly, the molecular docking and dynamics analyses, while supported by 100-ns simulations assessing pose stability, relied on binding-energy thresholds and hydrogen-bonding patterns as indirect indicators of target engagement rather than experimentally determined binding constants. Future studies should also benchmark the predicted binding affinities of quercetin, kaempferol, and luteolin against clinically established reference inhibitors for each target, to further contextualize the computational findings reported here. Therefore, the five hub targets identified here should be regarded as candidate targets requiring further validation rather than established therapeutic targets. The CCK-8-guided selection of sub-toxic concentrations was designed to isolate transcriptional effects from cytotoxic confounding, but does not preclude the possibility that gene expression changes are partly secondary to sub-lethal metabolic stress. HT-29 was selected as a widely used and molecularly characterized human colorectal adenocarcinoma model for preliminary *in vitro* validation (41). Nevertheless, CRC is molecularly heterogeneous, and no single cell line can represent its full molecular spectrum (42, 43). Therefore, the observed reductions in HIF1A, IL6, JUN, MMP9, and TNF mRNA should be regarded as preliminary evidence limited to HT-29 cells. Future studies should confirm these findings in additional CRC cell lines with distinct molecular backgrounds, together with protein-level, functional, organoid, and *in vivo* validation. Accordingly, the current results should be regarded as preliminary transcriptional evidence rather than direct proof of anti-cancer activity or therapeutic efficacy. Finally, protein-level validation by Western blot and functional assays (proliferation, invasion, apoptosis) are necessary to confirm that transcriptional suppression translates to phenotypic effects, and *in vivo* experiments in orthotopic or patient-derived xenograft models will be required to establish therapeutic relevance in a whole-organism context.

## 5 Conclusion

**Figure 7:**
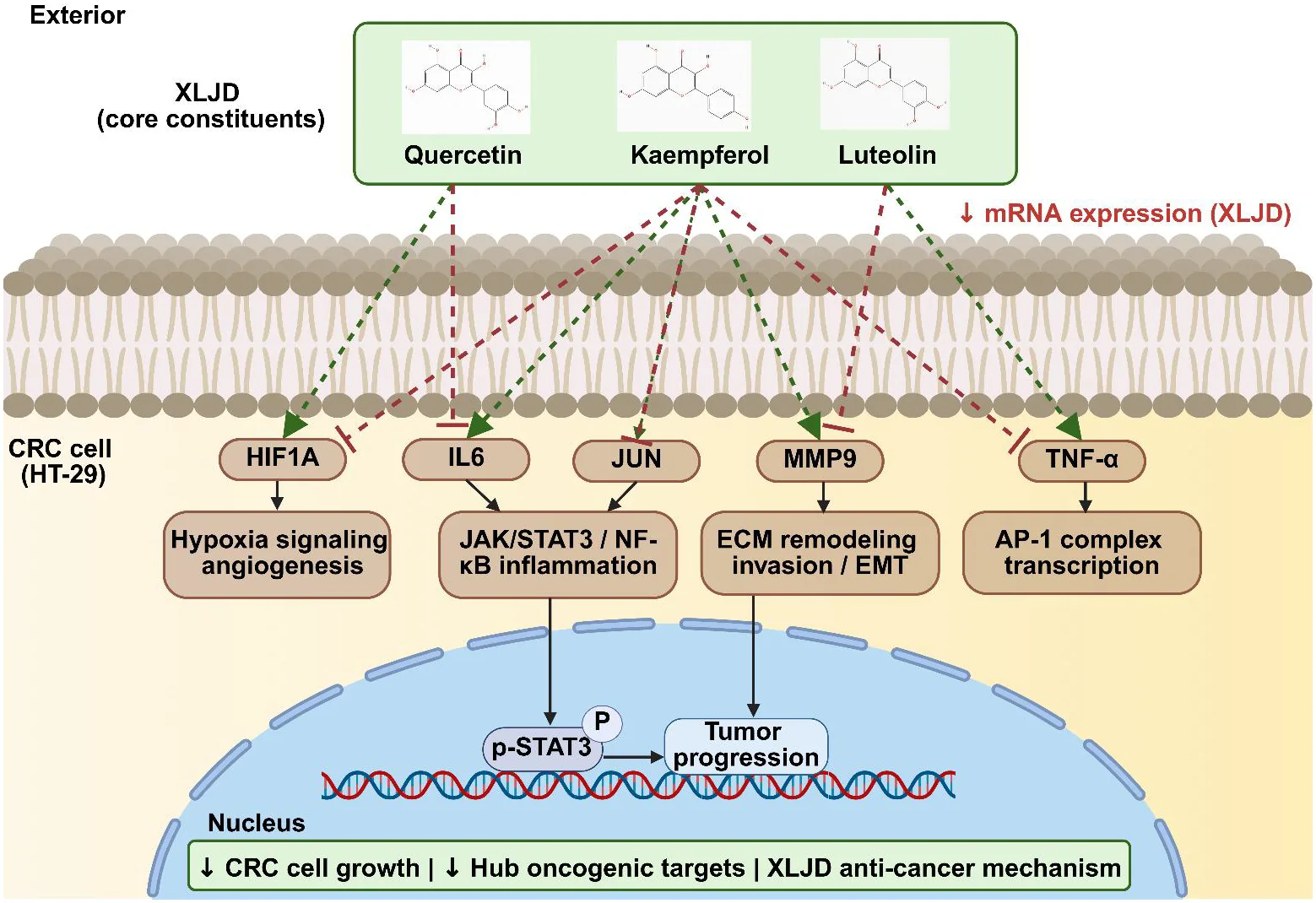
Proposed molecular mechanism by which Xian Lian Jie Du formula (XLJD) and its core active constituents exert anti-colorectal cancer effects. The three principal flavonoid constituents of XLJD, Quercetin, Kaempferol, and Luteolin, enter the colorectal cancer (CRC) cell and engage five hub oncogenic targets identified by network pharmacology and predicted by molecular docking: HIF1A, IL6, JUN, MMP9, and TNF-α. Green dashed arrows indicate direct binding interactions predicted by molecular docking; red dashed lines with T-bars denote transcriptional suppression of hub target mRNA expression, as validated by RT-qPCR in HT-29 cells. Downstream of these targets, four key oncogenic processes are modulated: hypoxia signalling and angiogenesis (via HIF1A), JAK/STAT3 and NF-κB inflammatory cascades (via IL6), ECM remodelling and epithelial-mesenchymal transition (via MMP9), and AP-1-mediated transcriptional activation (via JUN and TNF-α). Convergent downstream signalling is proposed to promote nuclear accumulation of phosphorylated STAT3 (p-STAT3) and drive tumour progression, consistent with established pathway biology; these downstream events warrant direct experimental validation in future studies. XLJD-mediated suppression of these pathways collectively results in inhibition of CRC cell growth, downregulation of hub oncogenic targets, and attenuation of the cancer toxin microenvironment, consistent with the formula’s traditional therapeutic principles of tonifying spleen qi, clearing heat and dampness, resolving blood stasis, and counteracting cancer toxin. P, phosphorylation; ECM, extracellular matrix; EMT, epithelial-mesenchymal transition; AP-1, activator protein-1.

This study combined network pharmacology, molecular docking, molecular dynamics simulation, and RT-qPCR to investigate the possible molecular basis of XLJD in colorectal cancer. HIF1A, IL6, JUN, MMP9, and TNF were identified as candidate targets linked to both XLJD and CRC. Treatment with XLJD and the selected flavonoids changed the mRNA expression of these genes in HT-29 cells, offering preliminary support for their involvement in the molecular effects of XLJD. Nevertheless, the experimental evidence was restricted to transcriptional changes in a single CRC cell line. Additional validation at the protein level, together with functional assays and *in vivo* studies, is therefore needed before the anticancer activity and clinical significance of XLJD can be established. A schematic summary of the proposed mechanism is provided above (**Figure 7**).

## Data Availability

The original contributions presented in the study are included in the article/**Supplementary Material**. Further inquiries can be directed to the corresponding authors.
