# Supplementary material for: Network pharmacology and experimental evidence of the anti-colorectal cancer potential of Xian Lian Jie Du formula: quercetin, kaempferol and luteolin jointly inhibit the HIF1A/IL6/TNF oncogenic pathway
Source: Front Oncol. 2026 Aug 31;16:1882054. doi: 10.3389/fonc.2026.1882054 (PMC13572154; doi:10.3389/fonc.2026.1882054)
Supplement: Supplementary file 1 [file Supplementaryfile1.docx]

**Supplementary Figure 1**

**Supplementary Figure S1.** Rg, SASA, and two-dimensional free-energy landscape analyses of all protein–ligand complexes during the 100-ns molecular dynamics simulations.

**Supplementary Figure 2**

**Supplementary Figure S2. Cell morphology of HT-29 cells treated at IC₅₀ concentrations.**

(A) HT-29 + 10% FBS; (B) HT-29 + 2.5% blank serum; (C) HT-29 + 5% blank serum; (D) HT-29 + 7.5% blank serum; (E) HT-29 + 10% blank serum; (F) HT-29 + 12.5% blank serum; (G) HT-29 + 15% blank serum; (H) HT-29 + 17.5% blank serum; (I) HT-29 + 20% blank serum; (J) HT-29 + 2.5% drug-containing serum; (K) HT-29 + 5% drug-containing serum; (L) HT-29 + 7.5% drug-containing serum; (M) HT-29 + 10% drug-containing serum; (N) HT-29 + 12.5% drug-containing serum; (O) HT-29 + 15% drug-containing serum; (P) HT-29 + 17.5% drug-containing serum; (Q) HT-29 + 20% drug-containing serum.

**Supplementary Figure 3**

**Supplementary Figure S3 Representative RT-qPCR amplification and melt curves.**
A–L show the amplification and corresponding melt curves of HIF1A, IL6, JUN, MMP9, TNF-α, and β-actin. Single melt peaks indicate good primer specificity.
